# Free Flaps in a Resource Constrained Environment: A Five-Year Experience—Outcomes and Lessons Learned

**DOI:** 10.1155/2015/194174

**Published:** 2015-08-11

**Authors:** Wanjala F. Nangole, Stanley Khainga, Joyce Aswani, Loise Kahoro, Adelaine Vilembwa

**Affiliations:** ^1^Department of Surgery (Plastic), University of Nairobi, P.O. Box 30197, Nairobi 00100, Kenya; ^2^Kenyatta National Hospital, P.O. Box 20723, Nairobi 00202, Kenya

## Abstract

*Introduction*. Free flap surgery is a routine procedure in many developed countries with good surgical outcomes. In many developing countries, however, these services are not available. In this paper, we audit free flaps done in a resource constrained hospital in Kenya. *Objective*. This is a five-year audit of free flaps done in a tertiary hospital in Kenya, between 2009 and 2014. *Materials and Methods*. This was a prospective study of patients operated on with free flaps between 2009 and 2014. *Results*. A total of one hundred and thirty-two free flaps in one hundred and twenty patients were performed during the five-year duration. The age range was eight to seventy-two years with a mean of 47.2. All the flaps were done under loupe magnification. The overall flap success rate was eighty-nine percent. *Conclusion*. Despite the many limitations, free flaps in our setup were successful in the majority of patients operated on. Flap salvage was noted to be low due to infrequent flap monitoring as well as unavailability of theatre space. One therefore has to be meticulous during surgery to reduce any possibilities of reexploration.

## 1. Introduction

Reconstruction of surgical defects requires a reconstructive surgeon to be well versed in all the reconstructive options. Simple defects could be reconstructed with the use of skin grafts or local flaps. Complex defects however require either regional, distant, or free flaps. Free flaps have been in use since inception about forty years ago [1–3]. Initially, they were practiced only in well established centres, though currently many centres in the developed countries routinely carry out these surgical procedures.

Successful free flaps surgeries require a well motivated and trained surgical team, good perioperative monitoring of the flaps, well equipped and readily available theatre space, good laboratory support services, and availability of intensive care unit beds [4]. The nursing team must also be ready to work for long hours without any reprieve.

The reality in many developing countries is however such that most of the above conditions are not available. Ironically, patients requiring free flap services are probably more than those in the developed countries (Figure 2). In this paper, we audit our work for the last five years in such an environment.

## 2. Materials and Method

### 2.1. Setting

The study was carried out at Kenyatta National Hospital, Nairobi, Kenya.

### 2.2. Study Design

This was a prospective study of patients reconstructed with free flaps.

### 2.3. Study Duration

Study duration was from August 2009 to December 2014.

### 2.4. Methodology

Patients reconstructed with free flaps at Kenyatta National Hospital were followed up for a minimum of six months. Data collected included patients' demographic features such as age and sex. Other information gathered included patients current and past medical conditions, anatomical location of the defect, and flaps used to reconstruct the defect (Tables 1 and 2). The surgical techniques employed for the patients during surgery were loupe magnifications for dissection and anastomosis of the vessels, arterial anastomosis before venous anastomosis, end-to-end arterial anastomosis with prolene 9/0 interrupted, end-to-end or end-to-side venous anastomosis with prolene 9/0, and topical application of heparin in a ratio of 100 units/mL of normal saline. Systemic heparin was occasionally utilized after the anastomosis. Postoperatively, patients were nursed in the critical care unit for at least 24 hours if a bed was available. Time taken after surgery and the first postoperative review of the flap and the frequency were noted. Intravenous antibiotics and clexane were given to all patients till the 7th postoperative day.

Flap related complications such as arterial compromise, venous congestion, haematoma, infections, and flap necrosis were documented. Donor site morbidity evaluated included wound dehiscence or skin-graft failure.

## 3. Results

A total of one hundred and twenty patients were operated on during the study duration of five years. The age range for the patients was eight years to seventy-two years with a median age of 51.4 years and a mean age of 47.2 years. The male-to-female ratio was three to two. Five patients were being managed for diabetes mellitus (4%) while six patients (5%) were HIV positive with one patient's CD4 count at less than 200. Seventy-three percent of the defects managed were in the head and neck region, fifteen percent lower limb, and seven percent upper limb.

Operatively, 16 flaps had two venous anastomoses done (turbocharged). The mean duration from the completion of surgery to the assessment of the flap was 10.35 hours. All flaps were assessed at least once per day in the first week of surgery. Fifteen percent were assessed at least twice per day in the first 24 hours of surgery. No flap was assessed at four-hourly interval. All the assessments were done with the use of the needle-prick technique. Of the fifteen flaps lost, eight flaps were lost due to venous congestion, five due to arterial occlusion, and two due to infections within one week after surgery. Reexploration was done in eight flaps with only one flap being salvaged. Haematoma was noted in five patients, four of whom had been given intravenous heparin during surgery.

Donor site morbidity was noted in 24 patients. Twenty-one patients had partial graft take that healed with wound dressings alone. These were ten patients with radial forearm donor site, seven patients with free fibula donor site, and four patients with anterior lateral thigh flap donor site. Three patients, one with free fibula donor site and two with anterior lateral thigh donor site, had a repeat skin graft to cover the donor site.

## 4. Discussions

Free flaps have revolutionized management of complex wounds. With the advent of free flaps, wounds that would otherwise not be managed are now routinely managed with good surgical outcomes [4–6]. Tumours that would otherwise be considered inoperable are now being operated on and defects reconstructed with free flaps. Microsurgery in many cases is now considered the first option in reconstructing complex defects, best in addressing form, function, and aesthetics.

**Figure 1:**
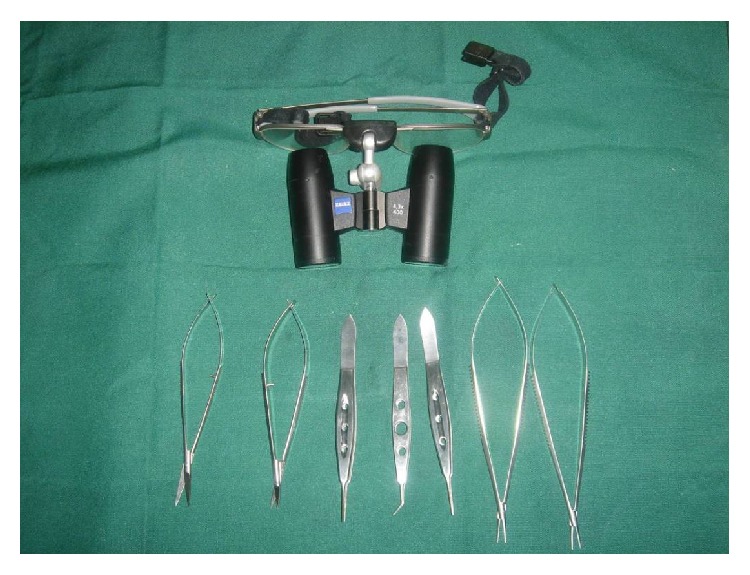
Basic instruments used for microsurgery.

**Figure 2:**
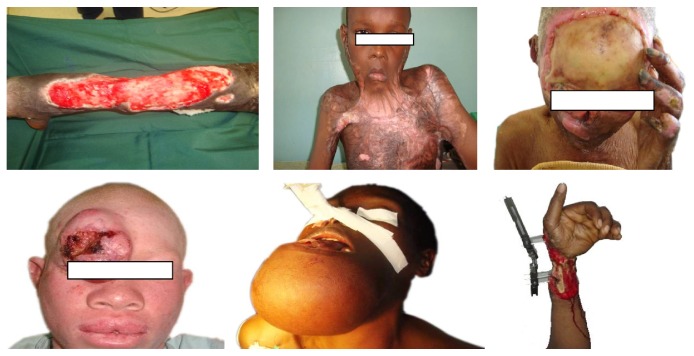
Multiple defects requiring free flaps for reconstruction in our hospital.

However, the practice of microsurgery has largely remained rudimentary if not nonexistent in many developing countries especially in sub-Saharan Africa. Few centres, if any, practice this important aspect of reconstructive surgery. In spite of this shortcoming, defects requiring free flaps are common; probably more than what is available in the developed countries (Figure 2). The reasons advanced for these are lack of surgical skills and necessary equipment to carry out the surgeries. However, as demonstrated in our series, free flaps could safely be carried out with the use of basic surgical equipment such as loupes which are readily available in many hospitals (Figure 1). Studies have also demonstrated using loupes for anastomosis to be just as effective as the microscope [7–9]. Anybody keen to perform free flaps could thus get basic training in microsurgery in any centre where it is frequently practiced. The best way to learn microsurgery is by practicing it.

**Figure 3:**
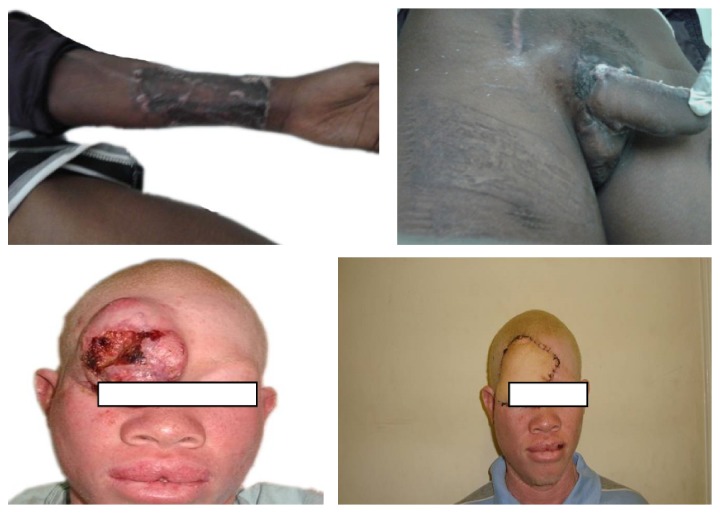
Radial forearm flap used for penile reconstruction and forehead reconstruction and its donor site.

Radial forearm flap in our series accounted for up to a third of the flaps done. The flap was commonly used for tongue reconstruction, penile reconstruction, and majority of the defects of the scalp and the face (Figure 3). It is a relatively easy flap to raise, has a long pedicle, and has relatively large veins allowing for ease of anastomosis. Among the disadvantages of the flap is the donor site morbidity that heals with extensive scarring even after grafting (Figure 3). This flap has been quoted in the literature as the gold standard for tongue, oral cavity, and penile reconstruction [10–12]. Free fibula flap accounted for almost twenty percent of the flaps done. The majority of these were for mandibular reconstruction (Figure 5). Free fibula flap is now considered the gold standard for mandibular reconstruction [13–17]. The skin paddle in our series was however noted to be unreliable in monitoring the flap.

**Figure 4:**
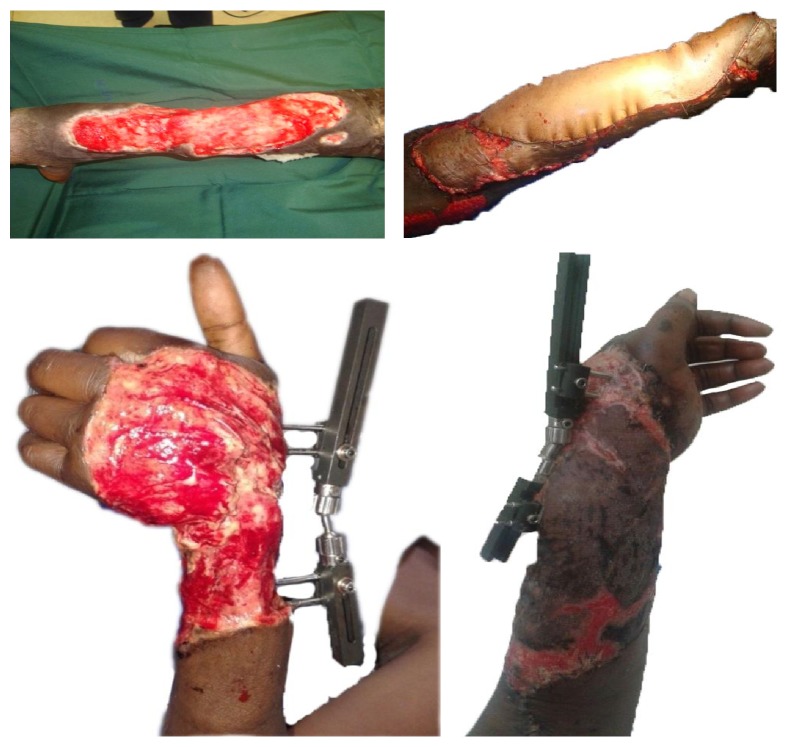
Latissimus dorsi flap utilised for reconstructing extensive defects of the extremities.

**Figure 5:**
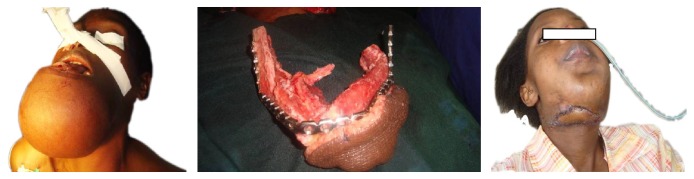
Free fibula flap for reconstructing mandibular defect.

**Figure 6:**
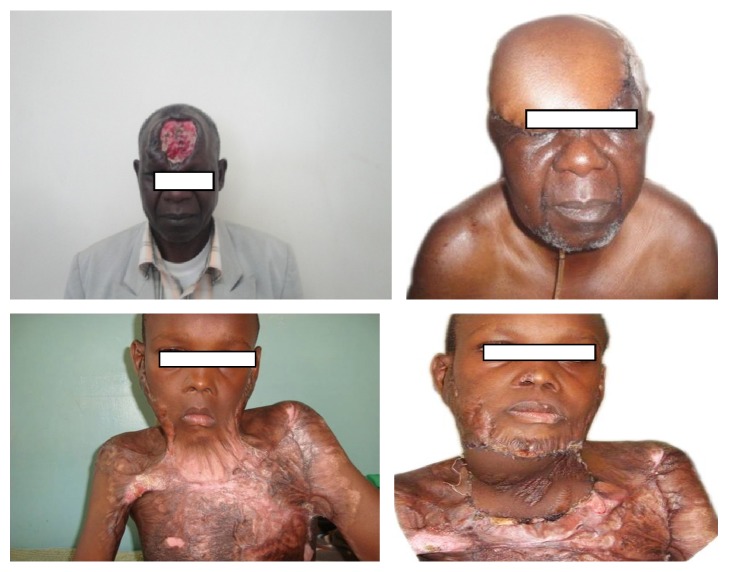
Anterior lateral thigh flap utilized in reconstructing extensive scalp and neck defect.

Latissimus dorsi flap was our workhorse flap for the defects of the extremities (Figure 4). This was either as a musculocutaneous flap or as a muscle flap. Among the advantages of this flap were the large surface area, long and reliable pedicle, and constant anatomical landmarks. The pedicle could also be raised with the nerve for functional reconstruction. It is probably rivaled by no other flap in the covering of extensive defects of the extremities [18–20]. Anterior lateral thigh flap was extensively used in our series for reconstructing large defects in the neck and the scalp region, with very good surgical outcome (Figure 6). The flap has been noted to be very effective for extensive soft tissue reconstruction especially in the head and neck region [5, 6, 21, 22]. The flap however has an unreliable perforator that even with the use of Doppler has to be searched for occasionally, extensively, before proceeding with the dissection. In two patients, the procedure had to be abandoned.

Perioperative monitoring of the flap was probably the biggest challenge in doing free flaps in our setup. Though currently there are many innovative ways of monitoring flaps that do not require the physical presence of the surgeon [23, 24], they are expensive and beyond the reach of our hospital. Monitoring was thus based on clinical examination and evaluation of the flap. Majority of the patients had their flaps reviewed for the first time almost twelve hours after surgery. This was due to a few number of staffs that could monitor the flaps, as well as the inability of the surgical team to do frequent monitoring. This probably explains the low flap salvage rate. To counter this, the operating team had to be extra cautious in ensuring flap survival, before reversing the patient from the operating table. Any slightest suggestion that the flap was not perfused well or was congested meant reexploring the anastomosis and starting over again. Another safety precaution taken was by using two venous anastomoses. This has now become our routine precaution measure in ensuring venous competence. Postoperative instructions were also clearly written on the patients dressings (i.e., because one flap was lost by the nursing team tying a dressing around the neck to secure a tracheostomy tube).

Haematoma formation in our patients was noted to be closely related with intravenous injection of heparin. This would later result in compression of the veins resulting in venous congestion. Of the eight flaps lost due to venous congestion, six of them were of patients on intravenous heparin. Routine use of intravenous heparin has since then been stopped and is only used in cases of difficult anastomosis or once a clot forms on the table. Topical irrigation of heparin on the other hand seems to be a safe procedure.

Though majority of our patients were HIV negative, our experience with patients who were HIV positive with low CD4 count was discouraging. In one such patient, one free flap was lost and one abandoned on the table after realizing poor recipient vessels with severe arteritis. A salvage pedicle flap had to be done. Those who do not get arterial or venous compromise are also more prone to wound sepsis, which can result in flap loss. Two of the three flaps lost due to sepsis were of patients who were HIV positive.

Our practice since then has been to be conservative for patients who are HIV positive.

In conclusion, free flaps are a viable option even in resource constrained environment. Good surgical outcomes could be realized by the use of basic surgical equipment such as loupes. The best approach is through a multidisciplinary approach with teams encompassing various related disciplines. The team must ensure adequate training in microsurgery before starting on the surgeries through fellowship programs or by inviting faculties from centres where these surgeries are done frequently. For starters, flaps that are relatively easy to raise and with good calibre vessels may be the best to start with, followed by more complex procedures. Meticulous surgical technique and careful observation of the flaps on the table and soon after surgery is paramount in ensuring good surgical outcome. The saying that “flaps are lost on the table” is probably truer in such an environment than anywhere else. The team must also learn to support each other in the event of flap failure and regroup again to try again, for that is the only sure way of being perfect with free flaps.

## Figures and Tables

**Table 1:** Summary of the surgical conditions the patients presented with.

Diagnosis	Frequency	Percentage
Cancer of the tongue	31	25.8
Mandibular defect	23	19.2
Neck contracture	8	6.7
Facial/scalp defects	21	17.5
Neck tumours	5	4.1
Leg defects	12	10
Upper limb defects	6	5
Upper limb lymphoedema	2	1.7
Lower limb lymphoedema	6	5
Palatal defects	3	2.5
Penile defects	3	2.5
Total	120	100

**Table 2:** Summary of flaps performed and the outcomes.

Flap performed	Frequency	Successful	Failed	Percentage successful
Radial forearm flap	48	43	5	89.5
Free fibula flap	25	22	3	88
Latissimus dorsi flap	19	18	1	94.7
Anterior lateral thigh	25	21	4	84
Parascapular flap	2	1	1	50
Gracilis muscle flap	1	0	1	0
Cervicofacial lymph node	12	12	0	100
Total	132	117	15	89
	100	89	11	
